# Neurophysiological mechanisms underlying interoceptive hypersensitivity in somatic symptom disorder: a mediation analysis of alexithymia

**DOI:** 10.3389/fpsyt.2026.1855147

**Published:** 2026-08-07

**Authors:** Cunyou Gao, Yujie Yuan, Xiaojun Zhuang, Yiqin Tang, Huanzhong Liu

**Affiliations:** 1 Department of Psychiatry, Chaohu Hospital of Anhui Medical University, Hefei, China; 2 Tongren Hospital, Shanghai Jiao Tong University School of Medicine, Shanghai, China

**Keywords:** alexithymia, electroencephalography (EEG), heartbeat-evoked potential (HEP), interoception, parallel mediation, somatic symptom disorder

## Abstract

**Background:**

Somatic Symptom Disorder (SSD) is defined by distressing physical symptoms and exaggerated psychological responses, and interoceptive hypersensitivity is widely regarded as a central feature, but the neuro-psychological pathway from neural interoceptive processing to clinical symptoms is not well elucidated. Hence, the present study sought to investigate the neurophysiological basis of interoceptive hypersensitivity in SSD and the mediating role of alexithymia.

**Methods:**

Fifty-two medication-naive patients with SSD and 52 age- and sex-matched healthy controls (HC) underwent clinical assessment with PHQ-15 and TAS-20, as well as high-resolution EEG recording, and interoceptive hypersensitivity was quantified via heartbeat-evoked potential (HEP) amplitudes obtained during a heartbeat tracking task and resting-state Phase Lag Index (PLI). A structural equation model (SEM) was then used to formally test the proposed parallel multiple mediation pathway.

**Results:**

SSD patients exhibited significantly higher somatic distress, alexithymia, and interoceptive accuracy compared to HCs (p< 0.001). Neurophysiologically, the SSD group demonstrated significantly increased resting-state PLI and higher HEP amplitudes (2.20 ± 1.10 vs. 1.20 ± 0.80 μV, p< 0.001), presenting a robust and clinically realistic effect size (Cohen’s d = 1.04). Mediation analysis confirmed that alexithymia significantly and specifically mediated the relationship between HEP amplitude and somatic symptom severity (specific indirect effect = 0.820, p = 0.025). A combined diagnostic model using HEP and TAS-20 scores achieved an Area Under the Curve (AUC) of 0.866.

**Conclusion:**

SSD is characterized by amplified cortical processing within the interoceptive network, and this neural hypersensitivity to visceral signals is strongly associated with clinical somatic distress, a process partially mediated by impaired emotional identification. Therefore, HEP amplitude and alexithymia are exploratory candidate indicators that directly support the use of neurophysiological and emotional regulation interventions in the treatment of SSD.

## 1 Introduction

Somatic Symptom Disorder (SSD), introduced in the fifth edition of the Diagnostic and Statistical Manual of Mental Disorders (DSM-5), marks a paradigmatic shift in the conceptualization of psychosomatic conditions. Departing from earlier diagnostic frameworks, such as those in DSM-IV, that centered on the “medically unexplained” nature of physical symptoms, DSM-5 redefines SSD around two core criteria (1): the presence of one or more distressing somatic symptoms, and (2) excessive, persistent thoughts, feelings, or behaviors related to those symptoms, including disproportionate concern about symptom seriousness, high levels of health-related anxiety, or time-consuming illness-related behaviors (1). In general hospital outpatient settings, particularly in China, epidemiological studies report prevalence rates of SSD ranging from 5% to 20%, yet clinical recognition remains low due to symptom overlap with medical conditions, lack of clinician training in functional disorders, and structural barriers to psychiatric consultation (2). This underdiagnosis contributes significantly to healthcare overutilization, recurrent specialist referrals, and repeated negative diagnostic workups—a self-perpetuating cycle that imposes substantial economic and psychological burdens on patients and systems alike (3, 4). Critically, the nosological transition from “somatoform disorders” to “somatic symptom disorder” reflects not merely semantic refinement but a substantive theoretical evolution: it replaces mind–body dualism with an integrative biopsychosocial model grounded in maladaptive illness behavior, attentional bias toward bodily signals, and emotion regulation deficits (5).

At the neurobiological level, emerging evidence positions interoceptive dysregulation as a central pathophysiological mechanism in SSD. Interoception, the neural process by which the brain senses, integrates, and interprets internal bodily signals (e.g., cardiac, respiratory, gastrointestinal), is essential for homeostatic regulation, emotional awareness, and embodied selfhood (6, 7). Contemporary predictive coding models, such as the Interoceptive Inference framework, posit that the brain continuously generates top-down predictions about internal states and updates them via bottom-up prediction errors (8). In SSD, this system appears biased toward heightened sensitivity to prediction errors, manifesting as “interoceptive hypersensitivity”: an amplified cortical response to routine physiological fluctuations (“biological noise”) that neurotypical individuals filter automatically (9). This aberrant signal weighting may drive misattribution of benign interoceptive input as threatening, thereby catalyzing symptom amplification and illness preoccupation.

To quantify this neural sensitivity objectively, the Heartbeat-Evoked Potential (HEP) has gained traction as a validated electrophysiological index of cortical interoceptive processing. The HEP is a time-locked cortical event-related potential peaking 200–600 ms after the R-wave of the electrocardiogram (ECG), reflecting cortical integration of cardiac afferent signals (10). Source localization and intracranial studies consistently implicate the posterior insula and anterior cingulate cortex (ACC) as primary generators, key nodes of the salience network implicated in detecting behaviorally relevant stimuli and regulating affective responses (11, 12). Notably, while cardiovascular pathologies (e.g., atrial fibrillation, hypertension) are associated with attenuated HEP amplitudes, suggesting impaired signal transmission, elevated HEP amplitude in SSD has been interpreted as a neurophysiological signature of hyper-vigilant interoceptive monitoring, potentially reflecting excessive allocation of attentional and computational resources to cardiac signals (13, 14).

However, neural hypersensitivity alone is insufficient to explain the emergence of clinically significant somatic symptoms; psychological moderators are indispensable. Alexithymia, a stable personality trait characterized by difficulties identifying and describing emotions, externally oriented thinking, and limited imaginative capacity, represents one such critical moderator (15). Meta-analytic and longitudinal data confirm its high prevalence in SSD (60–80%) and demonstrate that alexithymia severity independently predicts symptom chronicity, functional impairment, and treatment resistance (16). Crucially, alexithymia may impair the cognitive-emotional scaffolding required to contextualize interoceptive signals: individuals with elevated alexithymia show reduced differentiation between autonomic arousal (e.g., tachycardia) and discrete emotional states (e.g., anxiety), increasing the likelihood that amplified bodily sensations are misinterpreted as evidence of organic disease, a core mechanism of somatization (17, 18).

Despite strong theoretical convergence among interoceptive neurobiology, alexithymia, and somatization, empirical integration remains fragmented. Prior studies have examined brain–heart coupling in anxiety or depression, but few have tested whether alexithymia mediates the relationship between objective interoceptive neural signatures (e.g., HEP amplitude) and clinical somatic symptom burden in SSD (19). For instance, although population-based research links high alexithymia in adolescents to increased somatic complaints, partially mediated by depressive symptoms, the absence of concurrent neurophysiological measures leaves the causal chain from neural signal to subjective experience unresolved (20). Most importantly, prior studies exploring the psychological modulation of somatic symptoms have frequently failed to disentangle the specific mechanistic role of alexithymia from highly comorbid traits, such as general negative affect (anxiety and depression) or broader personality dimensions like neuroticism (21, 22). This methodological confounding leaves a critical question unanswered: Is alexithymia a highly specific cognitive bottleneck translating neural interoceptive hypersensitivity into somatic distress, or is it merely a proxy for generalized emotional distress? Clarifying this specificity through competing pathway models, such as parallel multiple mediation via structural equation modeling, is pivotal for isolating specific indirect effects while controlling for shared variance among closely related clinical constructs (23, 24).

Building upon these gaps, the present study utilizes high-resolution EEG and a synchronized ECG-locking paradigm to explore the neurophysiological mechanisms of SSD. We primarily investigate task-evoked HEP amplitudes during a heartbeat tracking task to capture the specific cortical processing of interoceptive signals. Our primary hypotheses are threefold. First, that SSD patients will exhibit significantly increased HEP amplitudes compared to healthy controls, reflecting neural hypersensitivity, and this neurophysiological divergence will remain robust even after strictly controlling for concurrent baseline heart rate and generalized negative affect (anxiety and depression). Second, this neural response will correlate positively with the severity of both alexithymia and somatic symptoms. Third, in a parallel multiple mediation framework accommodating competing psychological constructs (anxiety, depression, and neuroticism), alexithymia will significantly and specifically mediate the relationship between cortical interoceptive processing and clinical symptom severity. By constructing this integrated model, we aim to provide a scientist-practitioner framework that moves beyond subjective reporting toward a biological understanding of the embodied mind in SSD.

## 2 Methods

### 2.1 Participants and ethical approval

The study cohort comprised 104 participants, including 52 medication-naive individuals diagnosed with Somatic Symptom Disorder (SSD) and 52 healthy controls (HC) matched for age and gender. Recruitment was conducted from January to December 2025. Two senior psychiatrists independently confirmed the diagnosis according to the criteria outlined in the Diagnostic and Statistical Manual of Mental Disorders, Fifth Edition (DSM-5). Inclusion in the SSD group specifically required a Patient Health Questionnaire-15 (PHQ-15) score of 10 or higher and the absence of any comorbid major psychiatric conditions, such as schizophrenia or bipolar disorder. Eligibility for all participants was further restricted to right-handed individuals aged 18 to 60 with normal or corrected-to-normal vision.

The study protocol received formal approval from the Ethics Committee of Shanghai Jiao Tong University School of Medicine Affiliated Tongren Hospital (Approval No. 2024-090). In accordance with the principles of the Declaration of Helsinki, written informed consent was obtained from all participants before their involvement in any study procedures.

### 2.2 Clinical and psychological assessments

A comprehensive battery of psychometric instruments was utilized to profile the clinical and psychological status of the participants. Somatic symptom burden was quantified via the PHQ-15 and the Somatic Symptom Disorder-12 (SSD-12). Alexithymia was evaluated using the Toronto Alexithymia Scale (TAS-20), focusing on its three core dimensions: difficulty identifying feelings, difficulty describing feelings, and externally oriented thinking. Complementary assessments included the Self-Rating Anxiety Scale (SAS) and the Self-Rating Depression Scale (SDS) for emotional distress, alongside the Neuroticism subscale of the Eysenck Personality Questionnaire (EPQ-N) for broader personality profiling. These distinct psychological constructs were comprehensively assessed to subsequently test their specific competing roles in mediating somatic symptoms. While the SSD-12 was utilized to capture the maladaptive psychological criteria (Criterion B) of the disorder, the PHQ-15 was specifically selected as the primary dependent variable in our mediation models because it directly quantifies the physical severity of the somatic symptom burden itself (Criterion A).

### 2.3 Interoceptive task: the heartbeat tracking paradigm

Interoceptive accuracy was gauged through a modified Schandry heartbeat tracking task. Participants were positioned in a sound-attenuated, dimly lit environment and tasked with silently counting their heartbeats during three discrete, randomized intervals (e.g., 60, 90, and 150 seconds). On-screen prompts signaled the initiation and termination of each trial. To ensure the count relied solely on internal sensations, participants were strictly prohibited from physical pulse-taking or utilizing external aids. Concurrent electrocardiogram (ECG) recordings provided the objective heartbeat count. The Interoceptive Accuracy (HPS Score) was derived as follows:


HPS=1/3∑(1−|Recorded−Counted|Recorded)

where higher scores reflect a greater degree of interoceptive sensitivity.

### 2.4 EEG data acquisition

Electrophysiological signals were captured using a 64-channel Brain Products system (Brain Products GmbH, Germany), with Ag/AgCl electrodes arranged according to the International 10–20 system. Data were sampled at 1000 Hz with a hardware filter range from DC to 100 Hz, while electrode impedances were kept strictly below 5 kΩ. To facilitate the R-peak detection necessary for HEP analysis, two dedicated electrodes recorded ECG in a Lead II configuration. The recording protocol spanned a 10-minute resting state (5 minutes each for eyes-closed and eyes-open conditions) and 5 minutes during the active heartbeat tracking task.

### 2.5 EEG data processing and HEP extraction

Offline preprocessing was executed via EEGLAB and the FieldTrip toolboxes. Continuous data underwent band-pass filtering (0.5–40 Hz) and were re-referenced to the average of the bilateral mastoids. Independent Component Analysis (ICA) was systematically applied by a researcher blinded to group allocation to identify and suppress non-neural artifacts. Components reflecting ocular movements, muscular activity, and the cardiac field artifact (CFA) were rejected based on a standardized visual inspection of their scalp topographies and time courses. Adequate CFA removal was specifically confirmed by examining the absence of ECG-synchronized signal contamination in the retained EEG components.

To isolate the Heartbeat-Evoked Potential (HEP), ECG R-peaks were designated as time-zero. EEG epochs were extracted from -200 ms to 600 ms relative to the R-peak, with baseline correction performed using the -200 ms to 0 ms interval. Epochs with amplitudes exceeding ±100 μV were discarded. To explicitly rule out the possibility that differences in signal-to-noise ratio (SNR), potentially driven by varying heart rates or residual CFA, might confound the results, the total number of valid epochs retained after stringent preprocessing was extracted and statistically compared between the two groups. For each participant, valid trials were averaged to construct the HEP waveform. Following established literature, the mean HEP amplitude was calculated within a 200–400 ms post-R-peak window, a period identified as reflecting the most significant divergence in interoceptive processing between groups.

### 2.6 Statistical analysis

Statistical computations were performed using IBM SPSS Statistics 25 and R (version 4.5.1), with normality verified via the Shapiro-Wilk test. Group-wise comparisons for demographic, clinical, and electrophysiological data were conducted using independent sample t-tests or Chi-square tests for categorical variables. To rigorously exclude the confounding effects of autonomic arousal and negative affect on neural responses, a univariate Analysis of Covariance (ANCOVA) was applied to the evoked HEP amplitudes, incorporating resting heart rate, SAS, and SDS scores as covariates.

To robustly test the specificity of the neuro-psychological pathway, we upgraded the traditional mediation approach to a parallel multiple mediation model using the lavaan package. The model designated evoked HEP amplitude as the independent variable (X) and PHQ-15 score as the dependent variable (Y), while simultaneously entering TAS-20, SAS, SDS, and EPQ-N as parallel competing mediators. To optimize model fit and reflect the intrinsic correlations among these psychological constructs in clinical settings, their residual covariances were freely estimated. The significance of the specific indirect effects was assessed through 5000-iteration bootstrap sampling, which yields robust standard errors and ensures model stability even with moderate sample sizes. Additionally, a Receiver Operating Characteristic (ROC) curve analysis was performed using the pROC package to determine the diagnostic utility of the integrated neuro-psychological markers (combining evoked HEP amplitude and TAS-20 scores).

## 3 Results

### 3.1 Participant enrollment and characteristics

**Figure 1:**
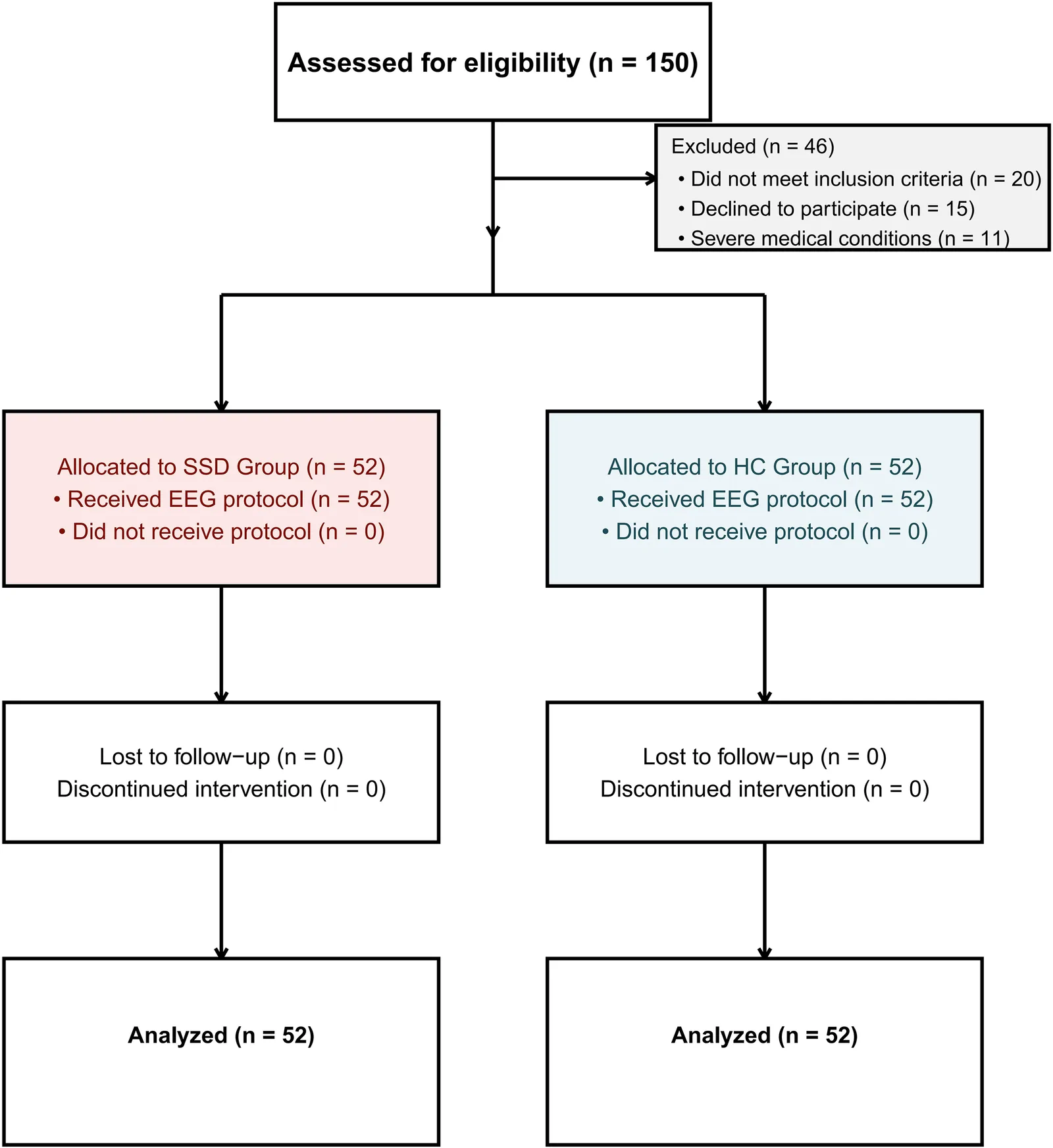
CONSORT flowchart of participant enrollment. The diagram illustrates the recruitment process and participant flow throughout the study. A total of 150 individuals were initially assessed for eligibility. Forty-six candidates were excluded based on pre-defined criteria (n=20), refusal to participate (n=15), or severe organic medical conditions (n=11). The final cohort of 104 participants was equally allocated into the Somatic Symptom Disorder (SSD) group (n=52) and the Healthy Control (HC) group (n=52). All enrolled participants completed the clinical assessments and EEG protocols without attrition.

Adhering to the CONSORT statement, the participant screening and enrollment process is detailed in **Figure 1**. Of the 150 candidates initially assessed for eligibility, 46 were excluded due to unmet inclusion criteria (n=20), refusal to participate (n=15), or the presence of severe organic medical conditions (n=11). The final analysis thus included 104 participants, comprising 52 patients with Somatic Symptom Disorder (SSD) and 52 healthy controls (HC), all of whom completed the full EEG protocol and clinical assessments without attrition.

Crucially, to ensure that electrophysiological group differences were not confounded by signal-to-noise ratio or trial count discrepancies (e.g., due to excessive cardiac field artifacts in the SSD group), the average number of valid epochs retained for HEP analysis was compared. Following strict ICA-based artifact rejection, the retained epochs did not differ significantly between the SSD group (139.8 ± 15.6) and the HC group (142.9 ± 15.0, *t* = -1.03, *p* = 0.305), confirming the high reliability and comparability of the EEG extractions.

Demographic and clinical baseline data for both groups are summarized in **Table 1**. No significant differences were found between the SSD and HC groups regarding age (40.85 ± 9.42 vs. 41.60 ± 8.74 years, *p* = 0.675) or years of education (13.54 ± 2.73 vs. 14.04 ± 2.72 years, *p* = 0.352), confirming successful demographic matching. Regarding clinical presentation, the SSD group demonstrated markedly higher levels of somatic distress (PHQ-15: 15.50 ± 6.55 vs. 1.37 ± 2.20, *p* < 0.001) and more severe psychological features characteristic of SSD (SSD-12: 23.27 ± 7.47 vs. 6.10 ± 4.49, *p* < 0.001). Furthermore, alexithymia scores were significantly elevated in the SSD group (TAS-20: 55.98 ± 11.97 vs. 41.92 ± 9.02, *p* < 0.001). For emotional states, SSD patients reported higher levels of anxiety (SAS) and depression (SDS) (*p* < 0.001), while their resting heart rate was slightly elevated compared to controls (77.99 ± 11.69 vs. 71.31 ± 8.57 bpm, *p* = 0.001).

**Table 1:** Demographic and clinical baseline characteristics.

Variable	HC (n = 52)	SSD (n = 52)	P-value
Age (years)	41.60 (8.74)	40.85 (9.42)	0.675
Gender (Female proportion)	0.54 (0.50)	0.63 (0.49)	0.324
Education (years)	14.04 (2.72)	13.54 (2.73)	0.352
PHQ-15 Score	1.37 (2.20)	15.50 (6.55)	<0.001
SSD-12 Score	6.10 (4.49)	23.27 (7.47)	<0.001
TAS-20 Score	41.92 (9.02)	55.98 (11.97)	<0.001
SAS Score	37.46 (5.58)	55.75 (8.04)	<0.001
SDS Score	40.46 (6.39)	56.60 (9.34)	<0.001
EPQ-N Score	47.19 (10.80)	48.79 (9.48)	0.425
Baseline Heart Rate (bpm)	71.31 (8.57)	77.99 (11.69)	0.001
Valid EEG Epochs	142.90 (15.02)	139.81 (15.59)	0.305

Data are presented as Mean (SD). The table summarizes demographic matching (Age, Education) and clinical symptom scores for the SSD (n=52) and HC (n=52) groups. Statistics include t-values from independent-sample t-tests, corresponding p-values, and Cohen’s d effect sizes. Additionally, the number of valid EEG epochs retained after artifact rejection is reported to confirm equivalent signal-to-noise ratios between groups. PHQ-15, Patient Health Questionnaire-15; SSD-12, Somatic Symptom Disorder-12; SAS, Self-Rating Anxiety Scale; SDS, Self-Rating Depression Scale; TAS-20, Toronto Alexithymia Scale; EPQ-N, Eysenck Personality Questionnaire-Neuroticism; HR, Heart Rate.

### 3.2 Behavioral, clinical, and resting-state neurophysiological profiles

**Figure 2:**
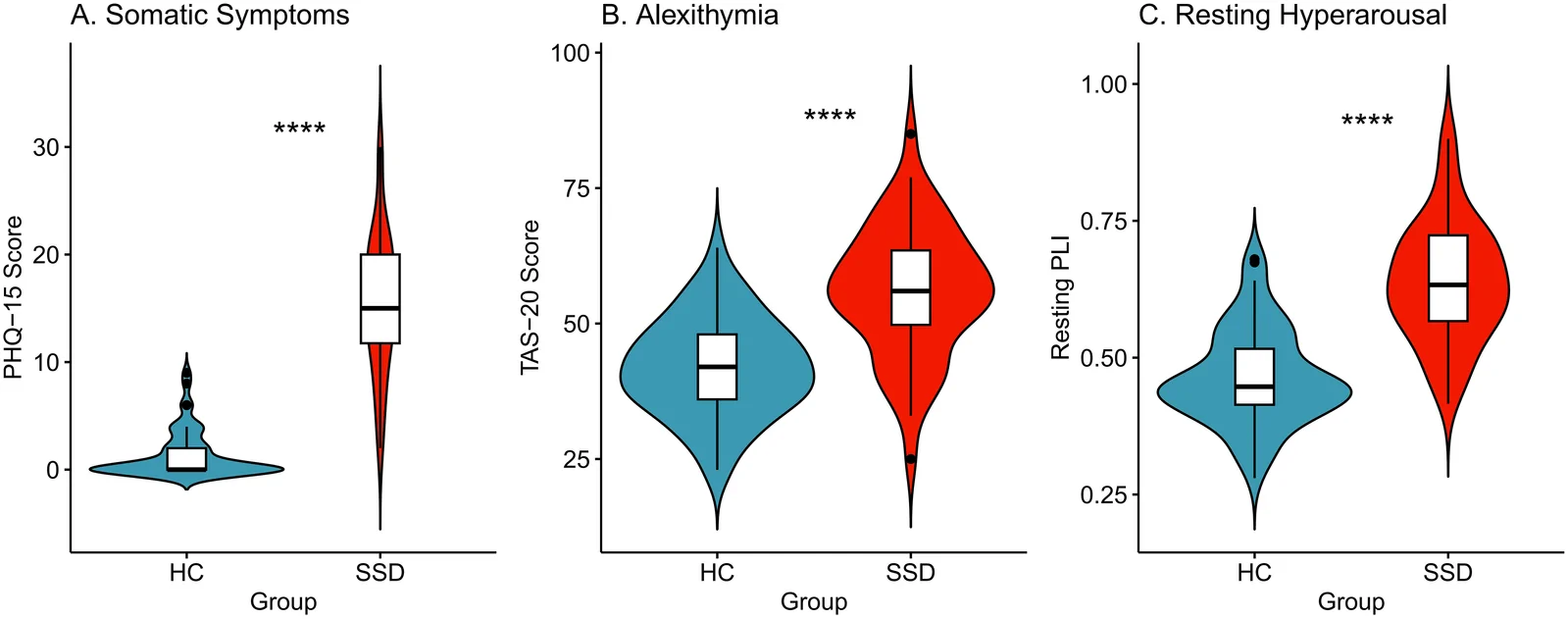
Multidimensional clinical and neurophysiological profile of SSD. Comparison of clinical and resting-state variables between SSD patients (red) and healthy controls (blue). **(A)** Somatic symptom severity as measured by the PHQ-15 score. **(B)** Alexithymia levels quantified by the TAS-20 score. **(C)** Baseline cortical hyperarousal is represented by the resting-state Phase Lag Index (PLI). Box-and-violin plots show the distribution, median, and interquartile range. Error bars represent the standard error of the mean (SEM). ****p< 0.001 based on independent sample t-tests.

To delineate the multidimensional pathological profile of SSD, we compared clinical, psychological, and resting-state electroencephalogram (EEG) indicators, as visualized in **Figure 2** and summarized in **Table 2**. Behaviorally, objective accuracy in the heartbeat tracking task (HPS score) was significantly higher in the SSD group than in healthy controls (0.73 ± 0.13 vs. 0.65 ± 0.14, *p* < 0.001), indicating pronounced interoceptive hypersensitivity.

**Table 2:** Behavioral and neurophysiological phenotypes of interoception.

Path or effect	Unstandardized estimate	SE	Standardized β	z-value	P-value
Direct paths (X → mediators, path a)					
Evoked HEP Amplitude → TAS-20 (a1)	2.667	1.121	0.227	2.378	0.017
Evoked HEP Amplitude → SAS (a2)	3.621	0.979	0.341	3.697	<0.001
Evoked HEP Amplitude → SDS (a3)	2.305	1.006	0.219	2.292	0.022
Evoked HEP Amplitude → EPQ-N (a4)	1.424	0.909	0.152	1.566	0.117
Direct paths (mediators → Y, path b)					
TAS-20 → PHQ-15 (b1)	0.308	0.044	0.454	6.93	<0.001
SAS → PHQ-15 (b2)	0.291	0.059	0.389	4.899	<0.001
SDS → PHQ-15 (b3)	0.069	0.054	0.091	1.281	0.2
EPQ-N → PHQ-15 (b4)	-0.045	0.048	-0.053	-0.926	0.355
Direct path (X → Y, path c')					
Evoked HEP Amplitude → PHQ-15 (c')	0.745	0.483	0.094	1.54	0.124
Mediation effects					
Specific Indirect Effect via TAS-20	0.82	0.365	0.103	2.249	0.025
Specific Indirect Effect via SAS	1.054	0.357	0.132	2.951	0.003
Specific Indirect Effect via SDS	0.159	0.142	0.02	1.118	0.264
Specific Indirect Effect via EPQ-N	-0.064	0.08	-0.008	-0.797	0.426
Total Effect (c)	2.715	0.734	0.341	3.699	<0.001

Comparison of interoceptive accuracy (HPS Score), resting-state functional connectivity (Resting PLI), and task-evoked neurophysiological response (Evoked HEP Amplitude). All metrics show significantly higher values in the SSD group (p< 0.001), supporting the interoceptive hypersensitivity hypothesis.

The distinct group separation in somatic symptoms (**Figure 2A**) and alexithymia scores (**Figure 2B**) further establishes the clinical and psychological divergence of the SSD cohort. This behavioral and psychological hypersensitivity was mirrored by resting-state EEG findings, where the SSD group displayed a significantly elevated Phase Lag Index (**Figure 2C**; Resting PLI: 0.64 ± 0.11 vs. 0.46 ± 0.09, *p* < 0.001). These results suggest that the brains of SSD patients exhibit cortical hyperarousal and abnormally enhanced functional connectivity, even in the absence of explicit interoceptive tasks.

### 3.3 Electrophysiological responses and correlations under evoked paradigm

**Figure 3:**
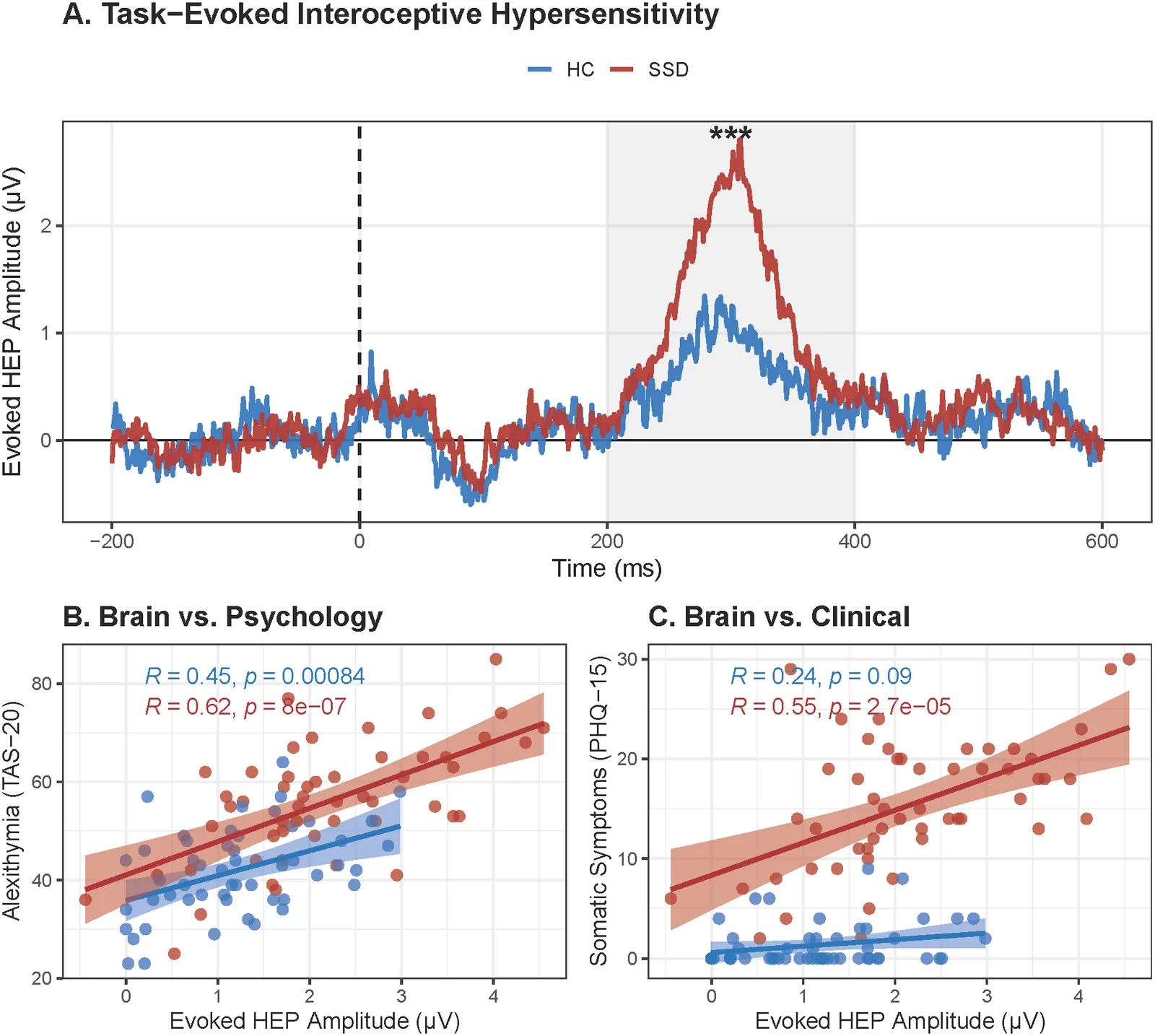
Task-evoked interoceptive hypersensitivity and brain-symptom correlations. **(A)** Mean amplitudes of the heartbeat-evoked potential (HEP) during the interoceptive task. SSD patients exhibited significantly higher HEP amplitudes compared to healthy controls (p< 0.001), with a robust effect size (Cohen’s d = 1.04). This group divergence remained highly significant in an ANCOVA model controlling for baseline heart rate, anxiety, and depression. **(B)** Correlation between evoked HEP amplitude and alexithymia (TAS-20 score), showing a significant positive association (R = 0.45, p< 0.001). **(C)** Correlation between evoked HEP amplitude and somatic symptom severity (PHQ-15 score; R = 0.55, p< 0.001). Shaded areas represent the 95% confidence interval of the regression line.

During the interoceptive evoked task, SSD patients demonstrated distinctly amplified electrophysiological responses compared to controls. As detailed in **Table 2** and the grand average waveform depicted in **Figure 3A**, the heartbeat-evoked potential (HEP) amplitude was significantly higher in the SSD group (2.20 ± 1.10 vs. 1.20 ± 0.80 μV, *p* < 0.001). This difference yielded a robust effect size (Cohen’s *d* = 1.04), indicating a substantial neurophysiological divergence in cardiac signal processing.

Importantly, given that HEP amplitude can be influenced by general autonomic arousal and negative affect, a univariate Analysis of Covariance (ANCOVA) was conducted. After concurrently controlling for resting heart rate (Baseline HR), anxiety (SAS), and depression (SDS) scores as covariates, the main effect of Group on evoked HEP amplitude remained highly significant (F (1, 99) = 16.24, p< 0.001). This suggests that the interoceptive neural hypersensitivity observed in SSD patients is a distinct pathophysiological correlate, rather than a mere secondary artifact of concurrent anxiety, depression, or tachycardia.

Correlation analysis further clarified the links between these evoked features and clinical dimensions. Scatter plots revealed that evoked HEP amplitude correlated significantly and positively with both alexithymia scores (**Figure 3B**; *R* = 0.45, *p* < 0.001) and somatic symptom severity (**Figure 3C**; *R* = 0.55, *p* < 0.001). These findings suggest that the intensity of abnormal cortical processing of internal signals is closely tied to psychological processing deficits and the overall degree of clinical somatic distress.

### 3.4 Parallel mediation model and diagnostic efficacy

**Figure 4:**
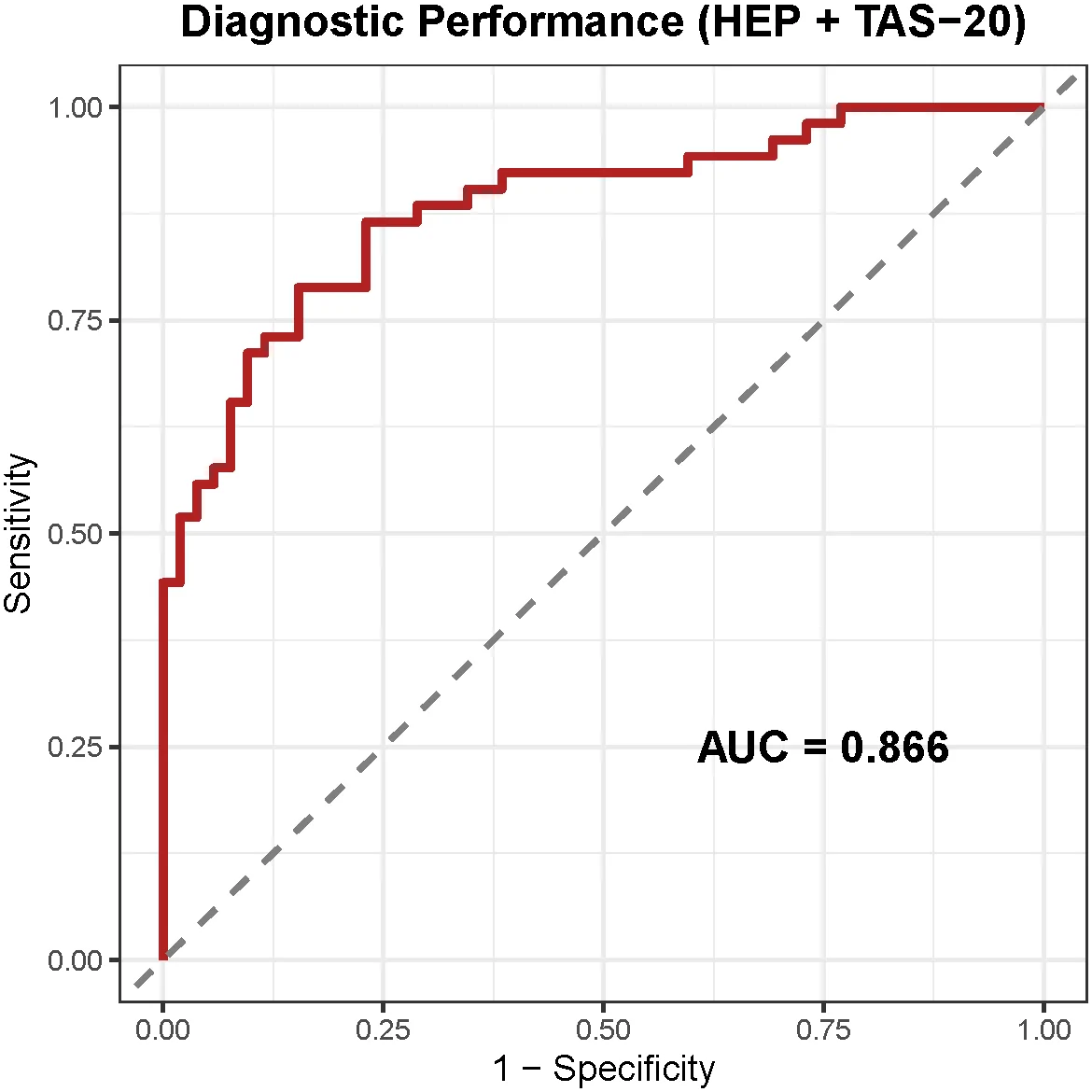
Parallel multiple mediation pathway. Structural Equation Model (SEM) path diagram illustrating the parallel mediating roles of distinct psychological constructs (TAS-20, SAS, SDS, and EPQ-N) bridging interoceptive neural processing and somatic symptoms. The diagram displays standardized path coefficients. The specific indirect effect via alexithymia (TAS-20) remained robustly significant, demonstrating its specific cognitive-emotional bottleneck role, whereas depression (SDS) and neuroticism (EPQ-N) did not yield significant mediating effects.

The final analysis established a psychological pathway model through structural equation modeling (SEM), connecting neural signals to clinical symptoms. To explicitly address the specificity of alexithymia as the core mediator, we expanded the analysis to a parallel multiple mediation model utilizing TAS-20, SAS, SDS, and neuroticism (EPQ-N) simultaneously as competing mediators (**Table 3**; **Figure 4**). After adjusting for these parallel competing constructs, Path a showed that HEP amplitude significantly predicted TAS-20 alexithymia scores (p = 0.017) and SAS anxiety scores (p< 0.001). In Path b, while anxiety (SAS) exhibited a parallel predictive effect on somatic distress, both depression (SDS, p = 0.200) and neuroticism (EPQ-N, p = 0.355) failed to yield specific indirect effects. Crucially, the specific indirect effect via TAS-20 remained highly robust and significant (p = 0.025). This parallel competing model strongly suggests that alexithymia is not just a general trait proxy for neuroticism or depression, but acts as a highly specific cognitive-emotional bottleneck mediating the translation of abnormal neural processing into clinical somatic symptoms.

**Table 3:** Path coefficients and parallel mediation effects in the SEM analysis.

Variable	HC (Mean ± SD)	SSD (Mean ± SD)	t-value	P-value	Cohen's d
Interoceptive Accuracy (HPS Score)	0.65 ± 0.14	0.73 ± 0.13	-3.4	<0.001	-0.67
Resting PLI	0.46 ± 0.09	0.64 ± 0.11	-9.04	<0.001	-1.77
Evoked HEP Amplitude (µV)	1.20 ± 0.80	2.20 ± 1.10	-5.3	<0.001	-1.04

Detailed results of the structural equation model showing the unstandardized estimates, standard error (SE), standardized beta (β), and p-values for each path. This table captures the competing indirect effects via four parallel mediators: TAS-20, SAS, SDS, and EPQ-N. Residual covariances among mediators were freely estimated to optimize model fit. Results confirm that alexithymia (TAS-20) and anxiety (SAS) function as significant specific mediators, while depression (SDS) and neuroticism (EPQ-N) do not.

**Figure 5:**
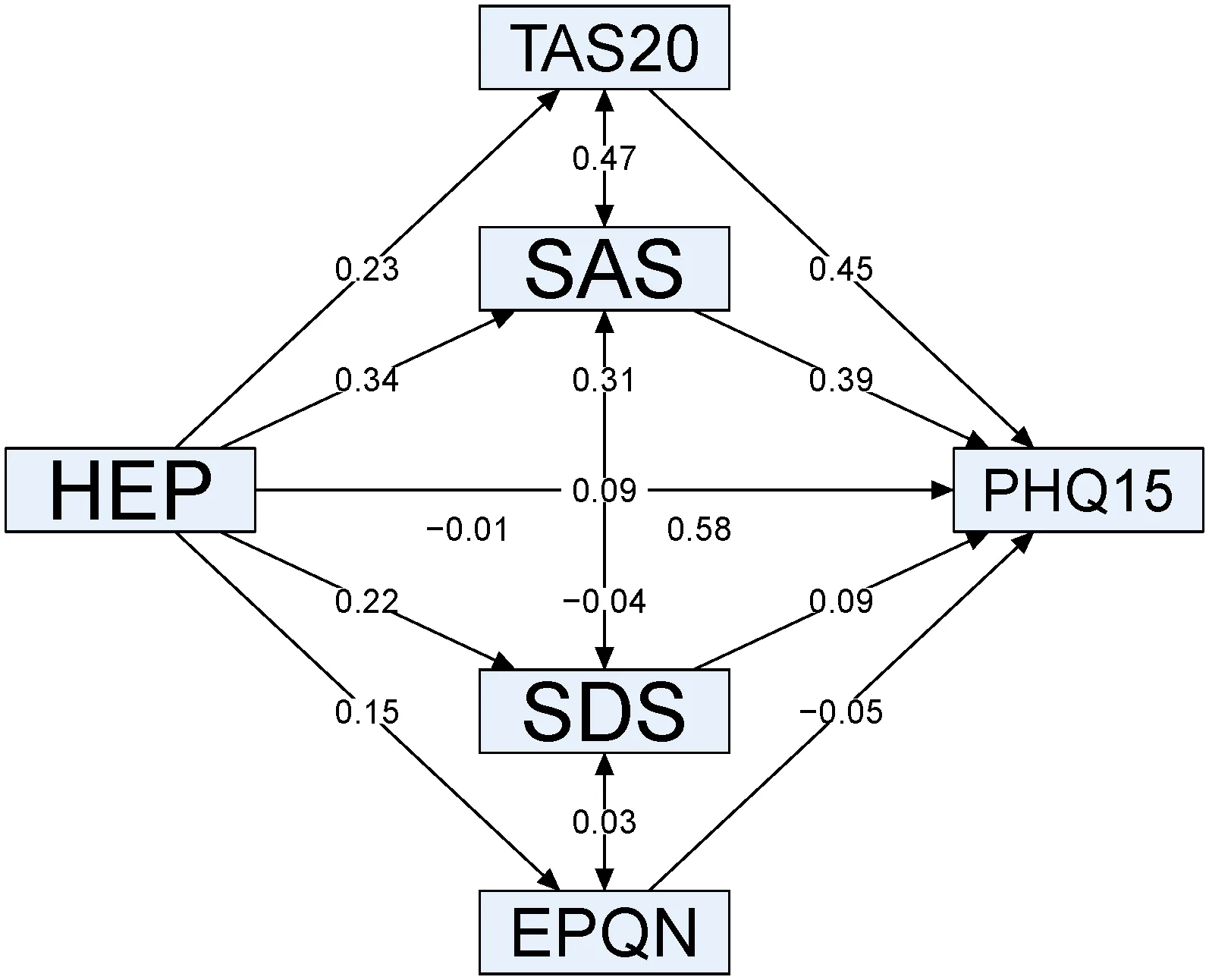
Diagnostic performance of integrated biomarkers. Receiver operating characteristic (ROC) curve analysis for discriminating SSD patients from controls using a combined model of evoked HEP amplitude and TAS-20 scores. The area under the curve (AUC) reached 0.866, indicating robust and clinically realistic diagnostic accuracy.

Lastly, we evaluated the clinical diagnostic potential of these integrated markers using ROC analysis. As shown in **Figure 5**, a diagnostic model combining evoked HEP amplitude and TAS-20 scores performed reliably in distinguishing SSD patients from healthy controls, yielding an Area Under the Curve (AUC) of 0.866. This suggests that neurophysiological indicators of interoception, when integrated with core psychological scales, provide exploratory support for their potential utility as candidate indicators in the assessment of Somatic Symptom Disorder.

## 4 Discussion

The present paper presents a well-specified, mechanistically rigorous neuro-psychological model of Somatic Symptom Disorder (SSD), combining high-resolution electrophysiological data with established psychological constructs. It indicates that SSD is characterized by a central interoceptive gain abnormality, as indicated by heightened task-evoked Heartbeat-Evoked Potential (HEP) amplitudes. The article presents a detailed analysis of the phase lag index (PLI) in cortical networks and then uses parallel multiple mediation analyses to show that alexithymia should be regarded not just as a comorbid trait or a proxy for general negative affect, but as an active and highly specific cognitive-emotional bottleneck that partially mediates the relationship between neural interoceptive hypersensitivity and clinical somatic symptom severity. Therefore, these findings frame SSD as a biologically grounded condition characterized by uncalibrated cortical representations of bodily signals lacking proper affective contextualization.

The elevation in task-evoked HEP amplitude (2.20 μV in SSD vs. 1.20 μV in healthy controls; Cohen’s d = 1.04, p< 0.001) constitutes electrophysiological evidence for pathological interoceptive amplification. Crucially, addressing potential methodological confounders, this neurophysiological hypersensitivity cannot be attributed to artifactual differences in signal-to-noise ratio or trial retention, as the valid EEG epochs were strictly matched between groups. Furthermore, our covariance analysis (ANCOVA) confirmed that this HEP elevation remains highly significant even after strictly controlling for resting heart rate, anxiety, and depression. This supports interoceptive hypersensitivity as a relevant pathophysiological trait rather than a secondary physiological byproduct of tachycardia or affective distress. This is because the HEP itself reflects the cortical integration of cardiac afferent input, with maximal source activity localized to the posterior insula and dorsal anterior cingulate cortex. These areas are well-established hubs of the salience network involved in detecting, evaluating, and prioritizing homeostatically relevant internal signals (10, 11). More importantly, the moderate-to-large effect size supports the conclusion that HEP amplitude is a highly sensitive and reliable index of interoceptive dysregulation in SSD, thus directly responding to long-standing calls for objective, quantifiable neurophysiological signatures in functional somatic disorders (13, 14).

The “hyper-HEP” phenotype is in contrast to the patterns seen in organic cardiovascular disease. Kumral et al. (2022) demonstrated attenuated HEP amplitudes in atrial fibrillation, which they linked to the disruption of temporal predictability caused by irregular R-R intervals, making cortical entrainment to cardiac signals impossible (13). Likewise, early-stage essential hypertension without structural brain changes also shows reduced HEP modulation. The association with reduced HEP modulation suggests a deficit in baroreceptor signal fidelity or a downstream thalamocortical gating abnormality (12). Hence, the various comparisons lead to a key distinction: whereas organic conditions typically involve signal degradation en route to the cortex, SSD represents a central gain enhancement, that is, a pathological upregulation of cortical weighting assigned to otherwise intact interoceptive inputs.

This aligns with the Interoceptive Inference framework, which proposes that SSD does not arise from faulty signal transmission, but rather from aberrantly high “precision-weighting” of prediction errors derived from visceral afferents. Hence, benign physiological fluctuations become perceptually salient and threatening (8, 14). The concurrent increase in resting-state PLI provides support for this view: it reflects a pre-existing state of heightened functional connectivity that predisposes the system to exaggerated task-evoked responses (19).

The translation of amplified neural interoceptive signals into clinically meaningful somatic distress is not automatic or inevitable, but rather requires higher-order affective processing capacity. This is why the parallel mediation model presented here shows that alexithymia functions as a critical psychological gateway. By placing alexithymia (TAS-20) in direct statistical competition with anxiety (SAS), depression (SDS), and neuroticism (EPQ-N) within a unified structural equation model, we answered critical methodological questions regarding construct specificity (21, 23). The results demonstrate that while anxiety also plays a parallel mediating role, neither depression nor neuroticism yielded significant specific indirect effects. This suggests that alexithymia is not merely a manifestation of generalized emotional distress or a neurotic personality profile (22, 24). Furthermore, this finding is fully consistent with both predictive coding and psychodynamic models: alexithymia impairs the ability to disambiguate overlapping autonomic and emotional cues (15, 17). Individuals with SSD who experience pathologically amplified interoceptive signals (indexed by high HEP) lack the conceptual scaffolding needed to interpret such arousal as context-appropriate emotion, so the uninterpreted signal defaults to a somatic attribution. “My heart is racing” becomes “Something is wrong with my heart”. This somatization process is best understood as a rational inference made by a system that is hyperattentive to bodily input but structurally under-equipped to assign emotional meaning to it (18). Therefore, the elevated PHQ-15 scores in our patients are not disordered but rather a coherent, neurocognitively constrained response: they exhibit amplified neural responses to bodily signals (high HEP) coupled with diminished emotional categorization capacities (high TAS-20), and hence experience intense distress.

The diagnostic performance of the integrated neuro-psychological model, with an area under the curve (AUC) of 0.866 for distinguishing SSD from healthy controls, provides a basis for rethinking SSD diagnosis. Rather than continuing to rely on the traditional “diagnosis of exclusion,” which is known to cause diagnostic delays, iatrogenic distress, and strain on the therapeutic alliance (2, 5), the authors advocate for a “diagnosis of inclusion”. Moreover, they identify a candidate replicable signature of SSD: elevated task-evoked HEP amplitude combined with elevated TAS-20 scores.

This conceptual shift has therapeutic implications: because the mediation analysis shows that alexithymia is a statistically significant moderator linking interoceptive hyper-reactivity to somatic burden, interventions targeting this mechanism have strong mechanistic plausibility. Evidence-based interventions such as interoceptive exposure training (to recalibrate cortical sensitivity) and emotion-focused therapy (EFT, to strengthen emotional labeling) therefore offer a path to attenuate the indirect pathway in the mediation model (6, 25). More importantly, by enhancing “emotional literacy,” these interventions can prevent the translation of amplified neural interoceptive input into disabling somatic distress, even before the underlying physiological signal itself is changed.

Several limitations merit careful consideration. First, the cross-sectional design precludes causal inference regarding the temporal ordering of neural and psychological alterations; longitudinal studies tracking HEP and TAS-20 trajectories from early symptom onset are needed to confirm developmental precedence (8). Second, while our ANCOVA and parallel mediation models successfully isolated interoceptive hypersensitivity from baseline anxiety and depression, this statistical control does not fully establish HEP specificity or complete independence from other unmeasured confounds, such as attention, vigilance, task engagement, respiratory variations, or residual cardiac field artifacts. Our combined diagnostic model was evaluated in a carefully selected, medication-naive cohort matched with healthy controls. Furthermore, while the Schandry heartbeat tracking task was employed as a classic behavioral paradigm, recent literature (27) highlights that performance may also reflect non-interoceptive top-down factors, such as time estimation abilities or prior beliefs about heart rate. Therefore, our behavioral metric should be interpreted cautiously as a composite of interoceptive attention and estimation strategies, which underscores the necessity of relying on our objective neurophysiological HEP data to draw conclusions. In highly complex, real-world psychiatric settings with substantial comorbidities (e.g., severe generalized anxiety or major depressive disorder), the discriminative specificity of this model requires further validation. Third, our paradigm focused exclusively on cardiac interoception. Whether this gain abnormality generalizes to other interoceptive channels (e.g., gastric motility or respiratory effort) represents a critical frontier for multimodal interoceptive profiling in SSD (26). Finally, future multimodal work integrating simultaneous EEG–fMRI in clinical populations will be essential to map the precise hierarchical circuitry (e.g., thalamo-insular–ACC pathways) underlying this gain dysregulation.

## 5 Conclusion

This paper gives a testable and biologically plausible account of the pathway underlying Somatic Symptom Disorder. SSD is closely associated with a central dysregulation of interoceptive gain control, which is quantifiably reflected in increased HEP amplitude that operates independently of resting cardiovascular tone and negative affect. Its clinical manifestation as distressing somatic symptoms (PHQ-15) is reliably gated by deficits in emotional processing, specifically alexithymia, which exhibits a specific mediating role distinct from general neuroticism or depression. Because the authors demonstrate actively measurable neural hypersensitivity rather than subjective fabrication, they properly establish the biological legitimacy of the suffering and therefore naturally lead to a precision psychiatry framework for psychosomatic conditions. More importantly, the findings go beyond validating patient experience by identifying a mechanistically well-defined, intervention-sensitive node: the interoceptive–affective interface. This interface can now serve as both a diagnostic anchor and a therapeutic target for next-generation embodiment-informed treatments.

## Data Availability

The original data supporting this study contain sensitive patient clinical information. In accordance with ethical review requirements and patient privacy protection principles, raw data are not publicly available. Qualified researchers can contact the corresponding author for reasonable data access requests, and de-identified research data will be provided after formal review and approval.
